# Determination of reference intervals for nonesterified fatty acids in the blood serum of healthy dogs

**DOI:** 10.1002/vro2.40

**Published:** 2022-07-25

**Authors:** Sophie‐Charlotte K. Doll, Peggy Haimerl, Alexander Bartel, Sebastian P. Arlt

**Affiliations:** ^1^ Clinic for Animal Reproduction Faculty of Veterinary Medicine Freie Universität Berlin Berlin Germany; ^2^ Institute for Veterinary Epidemiology and Biostatistics Faculty of Veterinary Medicine Freie Universität Berlin Berlin Germany

## Abstract

**Background:**

Nonesterified fatty acids (NEFAs) are an important energy substrate in mammals. Measurement of the NEFA concentration in blood serum is common practice and enables reliable detection of a negative energy balance in several species. This parameter can be used to detect subclinical metabolic diseases or to optimise feeding to prevent severe negative energy balance. Since no reference values for dogs have been published, the aim of this study was to establish such values.

**Methods:**

Blood serum from 85 healthy dogs was examined with a multiparameter clinical chemistry analyser. Given that NEFA values are not usually normally distributed, reference intervals (RIs) were calculated nonparametrically using bootstrapping (5000 replicates) for the 90% confidence intervals.

**Results:**

The examined cohort had a median age of 62.16 months (2–180 months) and a median weight of 19.2 kg (3.0–55.0 kg) and comprised 27 (31.8%) males and 58 (68.2%) females, with 32 (37.6%) neutered or spayed. The fasting time was 5.9 h (range 0–23 h). The tested confounders age, sex, neuter status, bodyweight and body condition score did not significantly affect the NEFA concentrations.

**Conclusions:**

The NEFA RI for dogs in this study was 0.2–1.47 mmol/L. The results may be used to adjust food composition and amount in healthy dogs or to detect metabolic disorders. Further research on NEFA metabolism in dogs maintained in standardised conditions and in specific nutritional situations or with particular diseases is warranted.

## INTRODUCTION

Adequate food supply (or feeding) plays a major role for dogs in terms of animal welfare and health. Food should be sufficient in amount and composition; it should be based on the actual demand regarding nutrients. However, studies have reported variation in nutritional demands due to individual differences in maintenance energy, which is used as a baseline measure based on weight, size, age, activity or other conditions, such as gestation and lactation.^1^, ^2^

Energy metabolism is controlled by a complex system of hormones, including adrenalin, noradrenalin, glucagon and adrenocorticotropic hormone. In times of scarcity or increased energy demand, glucose is depleted, and body fat is mobilised. Triglycerides, nonesterified fatty acids (NEFAs) and ketone bodies are released from fat tissue. In fasted polecats, the intra‐abdominal fat depots were reported to be mobilised more effectively than subcutaneous fat as a source of energy.^3^ NEFAs released from fat tissue are often bound to albumin and transported via blood circulation to target tissues,^4^ such as the liver or muscle, where they can be utilised.^4^, ^5^ Raised NEFA concentrations in the blood have been observed in fasted beagles, indicative of a negative energy balance.^6^

Some studies have reported that NEFA concentration is a valid parameter for diagnosing current energy deficiency in dairy cows.^7^, ^8^ There are several studies of NEFAs performed in carnivores such as polecats and beagle dogs.^6^ In obese polecats, NEFA concentrations were reported to have doubled after 5 days of fasting.^3^ Concentrations measured in blood serum are frequently used in daily practice to diagnose metabolic diseases in dairy cows and sheep.^9^ Most dairy cows experience a negative energy balance due to increased energy demand, decreased food intake and suboptimal composition of the ration around calving and early lactation.^10^

The use of NEFA concentrations has been described in horses. According to one study,^11^ feed‐restricted ponies had significantly higher NEFA concentrations than the control group. Mares are also at higher risk of developing postpartum colic when serum NEFA levels are increased.^12^ In dogs, the ketotic state usually develops in late gestation and lactation due to inadequate nutrition.^13^ In particular, the rapid fetal growth during the last 3 weeks of gestation and milk production in the first 4–6 weeks after parturition lead to a high energy demand.^13^, ^14^ While this may be related to an increase in NEFA concentration, to the best of the authors’ knowledge, this has not been reported. Consequently, the primary aim of this study was to determine the NEFA reference intervals (RIs) in a group of healthy dogs.

## MATERIALS AND METHODS

During routine visits at the Clinic for Animal Reproduction, Free University of Berlin, Germany or the veterinary practice Gemeinschaftspraxis Kreher, Stamnitz, Luckenwalde, Germany, owners of privately owned dogs were asked if they were willing to have their dog enrolled in the study. Participation of the dogs was given by informed consent of each owner. The project was reviewed and approved by the Landesamt für Gesundheit und Soziales Berlin, Germany (Reg 0165/16).

Altogether, owners of 113 dogs declared an interest in the study. Inclusion criteria were that the dogs had to be healthy, had not been under medical treatment during the 2 weeks prior to sampling and were at least 8 weeks old, irrespective of attributes such as breed, size, sex, stage of sexual cycle or sexual status (neutered or intact). Dogs showing signs of diseases during clinical examination or with reported diseases during the 2 weeks prior to sampling, dogs under medical treatment including long‐acting steroids or hormones, such as deslorelin, and dogs having leukocytosis were excluded. Leukocytosis was defined as a leukocyte concentration higher than 12.0 × 1000/μl.^15^

To ensure that dogs met the inclusion criteria, they were submitted to a clinical examination on the day of sampling. According to the anamnesis and the clinical examination, 104 clinically healthy dogs met the inclusion criteria and were eligible for blood sampling (Table 1). Specific data on the dogs, their medical history and feeding were recorded on case report forms developed for this study. Their body condition score (BCS) was determined following a five‐point scale.^16^ Bony prominences such as ribs, lumbar vertebrae and pelvic bones were assessed visually and via palpation. The status of the musculature, waist size and skeletal structure were assessed visually. According to their medical history, all dogs had been routinely treated with anthelmintics and had been regularly vaccinated according to national guidelines. For the study, dogs were not fasted, while their most recent food intake was documented according to owner statements. Bitches were neither lactating nor pregnant; the oestrous cycle stage of intact female dogs was not determined.

**TABLE 1:** Nonesterified fatty acid reference intervals (RIs) of 85 healthy dogs and different subgroups

		Reference interval (mmol/L)	
Group	Number of dogs	Lower reference limit (90% CI)	Upper reference limit (90% CI)
All dogs	85	0.20 (0.16‐0.24)	1.47 (1.04–1.71)
Sex			
Male	27	0.21 (0.16–0.21)	1.48 (1.48–2.21)
Female	58	0.17 (0.11–0.19)	1.64 (1.38–2.05)
Neuter status			
Neutered	32	0.16 (0.08–0.16)	1.48 (1.48–1.74)
Intact	53	0.20 (0.12–0.21)	1.72 (1.54–2.27)
Age			
<5 years	43	0.16 (0.1–0.16)	1.84 (1.78–2.51)
>5 years	42	0.21 (0.19–0.21)	1.47 (1.46–1.74)
Weight			
<16 kg	43	0.16 (0.08–0.17)	1.85 (1.81–2.5)
>16 kg	41	0.20 (0.15–0.2)	1.35 (1.35–1.53)

*Note*: The upper and lower RIs were calculated nonparametrically with 90% confidence intervals (CIs).

Blood serum and whole blood with ethylenediaminetetraacetic acid (EDTA) were used as sample material. Using a tourniquet, 4.0 ml blood was collected into a serum tube (Kabe Labortechnik, Nürnbrecht, Germany, 4 ml, polystyrol) and 1.0 ml into an EDTA tube (Kabe Labortechnik, 1 ml, polystyrol) from either the vena cephalica antebrachii or the vena saphena lateralis following the rules of good veterinary practice using a needle (21 G × 1½″ [0.8 × 38 mm] Terumo Agani Needle, Luer 6% Regular Bevel, Zhenjiang Kindly Medical Devices, Shanghai, China). During the general examination and sampling, the owners of the dogs stayed with their animal to attempt to minimise stress for the dog.

All serum tubes were centrifuged within 60 min after collection for 7 min at 2000× *g* (Hettich Zentrifuge EBA 20, Hettich, Tuttlingen, Germany). Subsequently, the cooled serum (6.0°C–8.0°C) was sent to a commercial laboratory (IVD Institut für Veterinärmedizinische Diagnostik, Berlin,Germany) via courier within 2 h.

The laboratory test ‘Non‐Esterified Fatty Acids’ (Randox Laboratories Ltd., Crumlyn, United Kingdom; accessed 5 July 2022) for quantitative detection for NEFAs on Cobas Mira Plus (F. Hoffmann‐La Roche Ltd., Rotkreuz, Switzerland) was used. Samples for NEFAs were measured in duplicate. If the results differed, the arithmetic mean was defined as the result. The test had (only) been internally validated by the laboratory for dog samples. For accuracy testing, standardised solutions were measured and spiked. The interassay coefficient of variation (CV) was 2.02% for the higher concentrated standard solution and 5.18% for the lower concentrated solution. The intraassay CV was 0.90% and 2.41%, respectively. The lower limit of detection (LoD) was 0.14 (mmol/L) when calculating LoD = 3.3*F*/*S*, where *S* is the slope of the calibration curve (estimation based on the calibration curve) and *F* is the calculated standard deviation by measuring replicates of a blank sample.

All statistical analyses were performed using R version 4.03 (R Foundation Vienna, Austria). Since NEFA values are lognormal distributed, a log_10_‐transformed scale was used for all figures. Calculation of RIs was performed in accordance with the American Society of Veterinary Clinical Pathology RI guidelines using the corresponding R package ‘Reference Intervals’ (version 1.2.0).^17^ Since NEFA values are not normally distributed, RIs were calculated nonparametrically using bootstrapping (5000 replicates) for the 90% confidence intervals (CIs). To visualise the continuous effect of weight, food intake and BCS on NEFA levels, ‘locally estimated scatterplot smoothing’ was used.

## RESULTS

Overall, 121 dogs of 39 different breeds were enrolled in the study. Of those, 13 had a medical history of recent illness (nine) or medication (four) and were, therefore, excluded. Another 19 dogs had to be excluded because of leukocytosis. Four samples were not suitable for analysis due to clotted EDTA blood. In total, 85 dogs remained for the statistical analysis (see Supporting Information S1). Of those dogs, 58 (68.2%) were female and 27 (31.8%) were male. The age of the dogs varied between 2 and 180 months, with a mean age of 62.16 months (5.18 years). The breed with the most individuals enrolled in the study was Dachshund, with 18 individuals, followed by four golden retrievers and four Swiss mountain dogs. All the other breeds were represented by one to three individuals. In addition, 16 dogs were classified as mongrels. For all dogs, 32 (37.6%) were neutered or spayed. The weight varied between 3.0 and 55.0 kg, with an average of 19.2 kg.

Based on our data, the upper reference limit for NEFAs in healthy dogs was determined as 1.47 mmol/L (CI 1.04–1.71 mmol/L). The lower reference limit was 0.20 mmol/L (CI 0.16–0.24 mmol/L).

**FIGURE 1:**
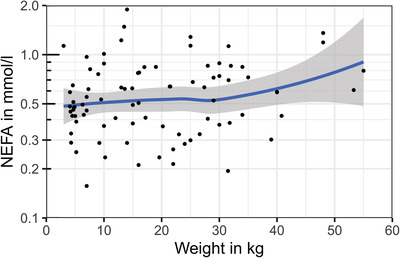
Nonesterified fatty acid (NEFA) concentrations of 85 healthy dogs in relation to bodyweight. Scatter plot of dog NEFA levels is expressed in mmol/L and the weight in kg. The *y*‐axis is log_10_‐transformed. The blue line shows the average effect of weight on NEFA levels and was calculated using ‘locally estimated scatterplot smoothing’, showing no significant correlation between the parameters

**FIGURE 2:**
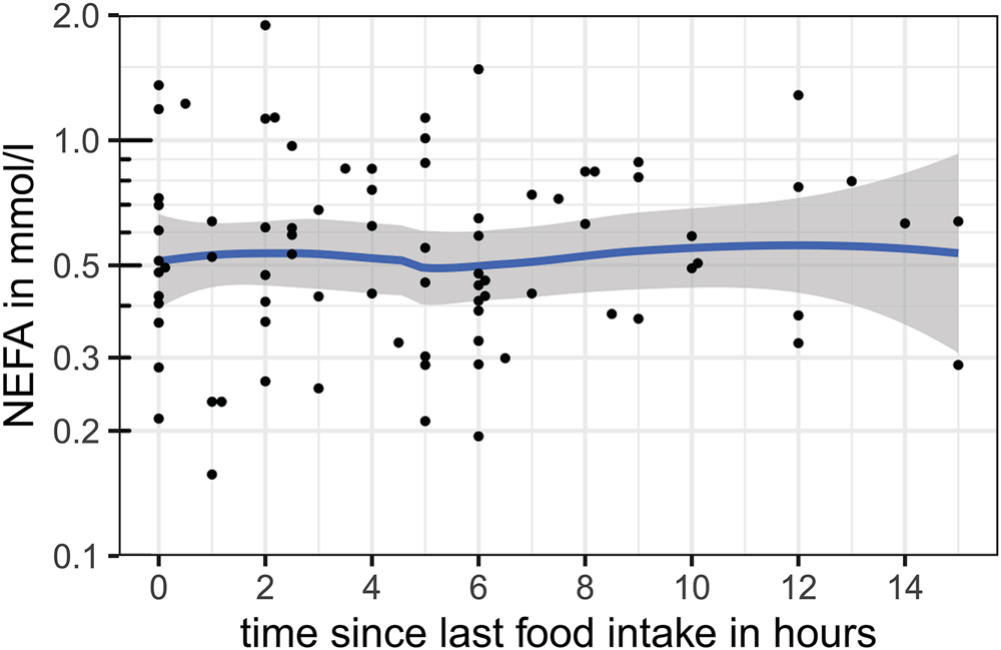
Nonesterified fatty acid (NEFA) concentrations of 85 healthy dogs in relation to the time interval from last food intake to sampling in hours. Scatter plot of dog NEFA levels in mmol/L and time interval from last food intake to sampling in hours. The *y*‐axis is log_10_‐transformed. The blue line shows the average effect of time since last food intake on NEFA levels and was calculated using ‘locally estimated scatterplot smoothing’, showing no significant correlation between the parameters

**FIGURE 3:**
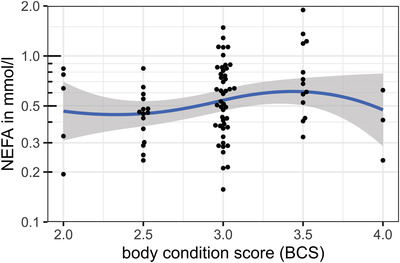
Nonesterified fatty acid (NEFA) concentrations of 85 healthy dogs in relation to body condition score (BCS). Scatter plot of dog NEFA levels in mmol/L and the BCS. The *y*‐axis is log_10_‐transformed. The blue line shows the average effect of BCS on NEFA levels and was calculated using ‘locally estimated scatterplot smoothing’, showing no significant correlation between the parameters

**FIGURE 4:**
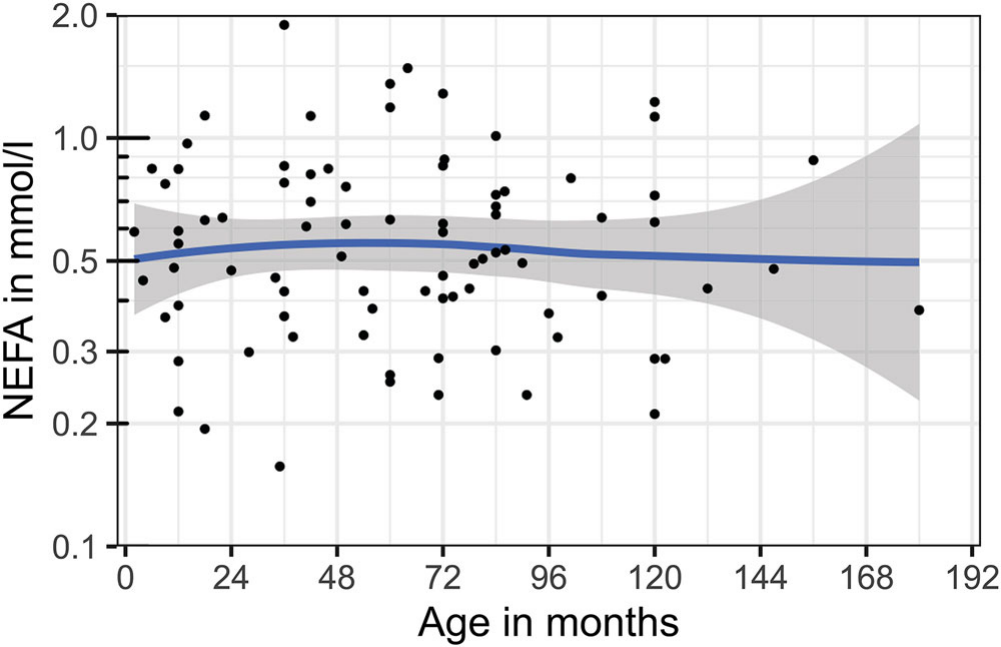
Nonesterified fatty acid (NEFA) concentrations of 85 healthy dogs in relation to age. Scatter plot of dog NEFA levels in mmol/L and age in months. The *y*‐axis is log_10_‐transformed. The blue line shows the average effect of age on NEFA levels and was calculated using ‘locally estimated scatterplot smoothing’, showing no significant correlation between the parameters

Different relevant confounders were tested for a possible effect on NEFA concentrations. It was tested whether sex, neuter status, age, bodyweight, breed, time since last feed uptake or BCS had a significant confounding effect. The listed parameters did not affect the NEFA concentrations, as shown in Table 1 and Figures 1, 2, 3, 4.

Nonesterified fatty acid concentrations seemed to slightly increase with weight (Table 1). Figure 1 shows that this effect was mainly based on four individuals with bodyweights of more than 45 kg. Nevertheless, single dogs of all weights showed higher NEFA concentrations, which could not be explained by long fasting or other metabolic factors considered in this study. The time interval from last food intake to sampling had no significant effect on NEFA concentrations in this study. Dogs with a BCS of 3.0 or 3.5 tended to have higher NEFA concentrations than dogs with lower BCS.

## DISCUSSION

To the best of the authors’ knowledge, this is the first study to report NEFA RIs for healthy dogs. NEFA concentrations may prove useful in the future as an additional tool to assess nutritional status in dogs.

The RI of 85 healthy dogs showed higher concentrations and a wider range compared to those defined for other species. In adult cattle and piglets aged 14 days, the NEFA RI was reported to range between 0.07 and 0.46 mmol/L and 0.05 and 0.18 mmol/L, respectively.^18^ In alpacas aged 6 months or younger, the NEFA RI was reported to lie between 0.16 and 1.6 mmol/L. In contrast, the NEFA values of alpacas older than 6 months were reported to have a much narrower range and lie between 0.1 and 0.7 mmol/L.^19^ In a study of obese beagle dogs, the NEFA concentrations before beginning a weight reduction programme were 0.36 ± 0.01 and 0.44 ± 0.04 for the two subgroups.^6^ These concentrations were within our RIs.

In our study, fasting time had no effect on NEFA concentrations. Only a few dogs, however, had fasted for more than 9 h and none for more than 15 h. It can be expected that longer fasting periods might have a significant effect on the measurement results. After prolonged fasting, blood metabolites, such as ketones and free fatty acids, may rise significantly in dogs.^20^ To assess the possible effects of fasting on NEFA concentrations, another study with longer and defined fasting periods would be necessary.

The study design could be defined as a ‘pragmatic trial’ with a convenience sample. Altogether, 39 different breeds were enrolled. This reflects a realistic dog population seen in daily veterinary practice. In our population, 18 dogs were Dachshunds. To avoid systematic bias, we also performed subgroup calculations regarding breed. As the Dachshund subgroup did not differ from the total data, a separate RI was not defined.

Analysis of the other subgroups showed no significant differences between them, although a larger sample size might have revealed small confounding factors present. Age, sex and neuter status had no influence on RI, which may mean that the parameter can be regarded as relatively robust against possible confounders. It must be considered that puppies and young dogs were not the focus of this study. Potentially, a study on the nutritional status and blood serum NEFA concentrations of puppies younger than 8 weeks may reveal different values for this parameter.

According to one study,^21^ growth hormone levels increase with weight and age, which may also have an influence on NEFA concentrations. The NEFA concentration in our study, however, only slightly increased with the weight of the tested dogs. This elevation was based on the data of only a few dogs and needs to be interpreted carefully. Furthermore, NEFA concentrations seemed to increase slightly with BCS, although these differences were not significant. As illustrated in Figure 2, dogs with a BCS of 3.5 had higher NEFA concentrations than those with BCS of 2.5 and 2.0. Only three sampled dogs had a BCS of 4.0, which showed lower NEFA concentrations compared to the cohort with a BCS of 3.5. Therefore, it remains open whether NEFA concentrations truly tend to increase with higher BCS. More dogs with a BCS of 4.0 or higher should be tested to assess a potential effect on the NEFA concentration.

In terms of physiology, the lower limit is certainly of less interest because in an anabolic state, the majority of NEFAs are stored within the adipocytes and can therefore only be measured at low concentrations. In that regard, further research should focus on the catabolic status, which is likely to result in a surplus of NEFAs. In human medicine, NEFA concentrations in combination with oral glucose tolerance tests can be used to detect changes in insulin sensitivity in pregnant women. This method is a novel approach and thus far not part of the routine diagnosis of many clinicians.^22^ In dogs, gestational diabetes is a potential application area for NEFA analysis because it can be tested using a laboratory test or a quick testing device usable on site.

One limitation of our study was the number of samples, as only 85 dogs met the inclusion criteria. Two of the subgroups formed, male dogs (*n* = 26) and neutered/spayed dogs (*n* = 32), had only a small number of samples. Certainly, our results must therefore be interpreted carefully. Ideally, at least 40 measurements should be performed for each subgroup.^17^

Another limitation was that dogs were not fed standardised diets. Enrolling more dogs belonging to only one breed under standardised housing, feeding and reproductive status would have potentially resulted in more precise data. However, having more variation in the sample pool better reflects the actual situation in veterinary practice.

We believe that blood NEFA concentration is a relevant parameter to measure negative energy balance in dogs. While the energy metabolism in dogs is significantly different from that in cattle, the measurement of NEFAs may potentially provide better insights into the actual energy supply in dogs compared to highly variable parameters such as glucose. Consequently, further studies are warranted of malnourished dogs or those with metabolic diseases.

## CONFLICTS OF INTEREST

The authors declare they have no conflicts of interest.

## FUNDING INFORMATION

The authors received no specific funding for this work.

## ETHICS STATEMENT

Ethical approval for the project was given by the Landesamt für Gesundheit und Soziales Berlin, Germany (Reg 0165/16).

## AUTHOR CONTRIBUTIONS

Sophie‐Charlotte K. Doll: Study design, collection of samples, statistical analysis, drafting the manuscript.

Haimerl, P.: Study design, collection of samples, proof reading.

Bartel, A: Study design, statistical analysis, drafting the manuscript.

Arlt, S: Study design, collection of samples, statistical analysis, drafting the manuscript.

## Supporting information

Supporting Information

## Data Availability

The data that support the findings of this study are available from the corresponding author upon reasonable request.
