# Supplementary material for: Determination of reference intervals for nonesterified fatty acids in the blood serum of healthy dogs
Source: Vet Rec Open. 2022 Jul 25;9(1):e40. doi: 10.1002/vro2.40 (PMC9313937; doi:10.1002/vro2.40)
Supplement: Supplementary file 1 — Supporting Information [file VRO2-9-e40-s001.docx]

**Supporting Information**

**Determination of reference intervals for nonesterified fatty acids in the blood serum of healthy dogs**

**Table S1**

Individual information of the dogs including breed, age, weight, sex, castration/neutering status, reason for consultation, leucocyte count, and NEFA concentration

| **Number** | **Breed** | **Age in months** | **Weight in kg** | **Sex** | **Neutered or spayed** | **Reason for presentation** | **Leucocytes Gpt/l (1000/µl)** | **Concentration of NEFAS (mmol/l)** |
| --- | --- | --- | --- | --- | --- | --- | --- | --- |
| 1 | Malinois | 56 | 31.8 | female | yes | vaccination | 6.1 | 0.382 |
| 2 | Mongrel | 72 | 25.0 | female | yes | health check | 9.6 | 1.284 |
| 3 | Mongrel | 180 | 15.0 | female | yes | health check | 11.6 | 0.379 |
| 4 | Rottweiler | 9 | 30.0 | male | no | health check | 11.5 | 0.007 |
| 5 | Pomeranian/ Bracke | 108 | 17.2 | female | yes | health check | 8.1 | 0.411 |
| 6 | Labrador x Great Dane | 18 | 31.5 | female | no | vaccination | 9.7 | 0.194 |
| 7 | Hannoverscher Schweißhund | 28 | 25.0 | female | no | vaccination | 11.6 | 0.299 |
| 8 | English Springer Spaniel | 12 | 17.0 | female | yes | health check | 8.1 | 0.839 |
| 9 | Labrador | 108 | 21.4 | female | yes | dental scaling | 4.5 | 0.638 |
| 10 | Golden Retriever | 72 | 30.0 | female | yes | cutting claws | 7.9 | 0.886 |
| 11 | Golden Retriever | 96 | 30.0 | male | yes | cutting claws | 9.2 | 0.372 |
| 12 | Mongrel | 132 | 6.5 | female | yes | health check | 11.6 | 0.428 |
| 13 | Mongrel | 6 | 19.0 | female | no | castration | 9.5 | 0.841 |
| 14 | Pomeranian x Terrier | 120 | 14.9 | male | yes | emptying anal glands | 10.3 | 0.622 |
| 15 | Mongrel | 91 | 19.6 | male | yes | vaccination | 7.7 | 0.235 |
| 16 | Jack Russel Terrier | 71 | 10.2 | female | yes | vaccination | 10.9 | 0.235 |
| 17 | Mongrel | 18 | 25.0 | female | no | vaccination | 9.9 | 1.134 |
| 18 | Jack Russel Terrier | 35 | 7.0 | female | yes | health check | 7.8 | 0.157 |
| 19 | Golden Retriever | 72 | 28.0 | female | no | health check | 7.0 | 0.855 |
| 20 | Giant Schnauzer | 12 | 40.0 | female | no | health check | 11.1 | 0.592 |
| 21 | Great Swiss Mountain Dog | 101 | 55.0 | male | no | health check | 9.7 | 0.797 |
| 22 | Rottweiler | 12 | 16.1 | male | no | health check | 10.1 | 0.777 |
| 23 | Jack Russel Terrier | 18 | 13.0 | male | no | castration | 11 | 0.629 |
| 24 | Weimeraner | 9 | 15.9 | male | no | castration | 9.6 | 0.772 |
| 25 | Schnauzer-Mix | 71 | 23.0 | male | yes | health check | 6.3 | 0.325 |
| 26 | German Shepherd | 46 | 34.0 | male | no | castration | 10.3 | 0.841 |
| 27 | Poodle | 84 | 10.0 | female | no | health check | 8.3 | 1.011 |
| 28 | Poodle | 156 | 10.0 | female | yes | health check | 5.9 | 0.882 |
| 29 | Poodle | 12 | 7.0 | female | no | health check | 10.7 | 0.551 |
| 30 | Toy Poodle | 41 | 3.0 | female | no | health check | 8.5 | 1.132 |
| 31 | Labrador | 120 | 29.0 | female | yes | health check | 8.7 | 0.723 |
| 32 | Wire-Haired-Dachshund | 14 | 7.0 | female | no | health check | 12 | 0.969 |
| 33 | Wire-Haired-Dachshund | 7 | 9.5 | female | no | health check | 8 | 0.532 |
| 34 | Wire-Haired-Dachshund | 50 | 7.3 | female | no | health check | 9.8 | 0.615 |
| 35 | Griffon Bleu | 5 | 27.4 | female | yes | health check | 7.2 | 0.631 |
| 36 | French Bulldog | 36 | 14.0 | female | no | ovulation timing | 11.1 | 1.891 |
| 37 | Great Swiss Mountain Dog | 60 | 48.0 | female | no | ovulation timing | 8.7 | 1.188 |
| 38 | Great Swiss Mountain Dog | 60 | 48.0 | female | no | ovulation timing | 10.6 | 1.357 |
| 39 | Labrador | 72 | 40.0 | male | yes | health check | 10.5 | 0.588 |
| 40 | Rhodesian Ridgeback | 66 | 40.8 | female | yes | health check | 8.9 | 0.422 |
| 41 | Dachshund-Mix | 90 | 6.3 | female | yes | health check | 5.3 | 0.494 |
| 42 | Golden Retriever | 120 | 31.6 | male | no | health check | 8.5 | 1.127 |
| 43 | Labrador x Border Collie | 9 | 20.2 | female | no | health check | 10.4 | 0.364 |
| 44 | Great Münsterländer-Mix | 42 | 31.6 | female | no | health check | 9.4 | 0.698 |
| 45 | Labrador x Poodle | 60 | 22.0 | male | yes | health check | 9.5 | 0.263 |
| 46 | Fox Terrier | 120 | 9.2 | female | yes | health check | 7.9 | 0.288 |
| 47 | Giant Schnauzer Mix | 84 | 30.0 | female | yes | health check | 7.7 | 0.740 |
| 48 | Kromfohrländer | 72 | 12.0 | female | no | ovulation timing | 9.3 | 0.409 |
| 49 | Kromfohrländer | 37 | 10.0 | female | no | health check | 8.8 | 0.366 |
| 50 | Dachshund | 68 | 4.5 | male | no | health check | 11.9 | 0.422 |
| 51 | Dachshund | 4 | 4.2 | female | yes | health check | 5.7 | 0.448 |
| 52 | Dachshund | 73 | 4.3 | male | no | health check | 11.2 | 0.289 |
| 53 | Dachshund | 54 | 4.3 | male | no | health check | 8.5 | 0.329 |
| 54 | Dachshund | 4 | 4.1 | female | yes | health check | 8.3 | 0.589 |
| 55 | Dachshund | 147 | 4.9 | female | no | health check | 7.9 | 0.478 |
| 56 | Hunting Terrier | 66 | 13.5 | male | yes | health check | 9.5 | 1.482 |
| 57 | Australian Shepherd | 87 | 25.4 | male | yes | health check | 7.4 | 0.680 |
| 58 | Border Collie | 10 | 14.0 | female | yes | health check | 6.1 | 0.288 |
| 59 | Labrador | 41 | 26.6 | female | yes | health check | 6.9 | 0.326 |
| 60 | Parson Terrier | 11 | 4.1 | female | no | health check | 10 | 0.481 |
| 61 | Dachshund | 13 | 5.2 | male | no | health check | 11.3 | 0.389 |
| 62 | Dachshund | 85 | 4.6 | male | no | health check | 10.1 | 0.649 |
| 63 | Dachshund | 70 | 4.7 | male | no | health check | 9.5 | 0.460 |
| 64 | Cocker Spaniel | 81 | 16.0 | male | no | health check | 8.1 | 0.506 |
| 65 | Cocker Spaniel | 79 | 15.0 | male | no | health check | 10.1 | 0.492 |
| 66 | German Shepherd | 84 | 39.0 | male | yes | health check | 5.1 | 0.302 |
| 67 | German Shepherd x Husky | 86 | 29.0 | female | yes | health check | 10.7 | 0.524 |
| 68 | Mongrel | 52 | 9.0 | female | yes | health check | 7.7 | 0.760 |
| 69 | Wäller | 12 | 24.0 | male | no | health check | 10.3 | 0.284 |
| 70 | Wäller | 12 | 22.0 | female | no | health check | 9.1 | 0.214 |
| 71 | Appenzeller Mix | 120 | 16.0 | male | yes | health check | 7.2 | 0.211 |
| 72 | Mongrel | 120 | 13.0 | female | yes | health check | 4.2 | 1.225 |
| 73 | Shorthair Collie | 73 | 28.0 | male | no | health check | 6.9 | 0.405 |
| 74 | Saarloos Wolfhound | 84 | 35.0 | female | no | health check | 6.8 | 0.607 |
| 75 | Great Swiss Mountain Dog | 41 | 53.3 | female | no | health check | 9.7 | 0.421 |
| 77 | Dachshund | 36 | 5.0 | female | no | health check | 9.5 | 0.428 |
| 78 | Doberman | 78 | 34.0 | female | no | health check | 11.1 | 0.854 |
| 79 | Doberman | 36 | 32.0 | female | no | health check | 11.4 | 0.513 |
| 80 | Dachshund | 49 | 4.7 | female | no | health check | 6.3 | 0.815 |
| 81 | Dachshund | 42 | 7.6 | female | no | health check | 10.1 | 0.474 |
| 82 | Dachshund | 24 | 4.8 | female | no | health check | 11.5 | 0.455 |
| 83 | Dachshund | 34 | 7.0 | female | no | health check | 7.3 | 0.617 |
| 84 | Krohmfortländer | 72 | 13.6 | female | no | health check | 10.3 | 0.253 |
| 85 | Dachshund | 60 | 5.2 | female | no | health check | 8.6 | 0.638 |
